# Multimorbidity and Polypharmacy in Chinese Emergency Department Patients With Atrial Fibrillation and Impacts on Clinical Outcomes

**DOI:** 10.3389/fcvm.2022.806234

**Published:** 2022-01-28

**Authors:** Juan Wang, Yan-min Yang, Jun Zhu, Han Zhang, Xing-hui Shao

**Affiliations:** Emergency and Intensive Care Center, State Key Laboratory of Cardiovascular Disease, Fuwai Hospital, National Center for Cardiovascular Disease, Chinese Academy of Medical Science and Peking Union Medical College, Beijing, China

**Keywords:** atrial fibrillation, multimorbidity, polypharmacy, vitamin K antagonist, outcomes, survival

## Abstract

**Background and Objects:**

Few studies focus on multimorbidity and polypharmacy in Chinese atrial fibrillation (AF) patients. We examined the impact of multimorbidity, polypharmacy, and treatment strategies on outcomes in Chinese emergency department (ED)AF patients. We also assessed factors associated with vitamin K antagonist (VKA) non-use in AF patients with multimorbidity or polypharmacy.

**Methods:**

2015 AF patients who presented to emergency department (ED) were enrolled from Nov 2008 to Oct 2011, mean follow-up of 12-months. Cox regressions were performed to identify the impact of multimorbidity and polypharmacy on clinical outcomes.

**Results:**

Six hundred and sixty-five patients in low morbidity group (≤1 comorbidity), 608 patients in moderate morbidity group (2 comorbidities), 742 patients in high morbidity group (≥3 comorbidities). Five hundred and seventy patients (28.3%) had polypharmacy (≥5 medications). High and moderate morbidity groups were significantly associated with a higher risk of all-cause death (*HR* 2.083, 95%*CI* 1.482–2.929; *HR* 1.713, 95%*CI* 1.198–2.449), CV death (*HR* 2.457, 95%*CI* 1.526–3.954; *HR* 1.974, 95%*CI* 1.206–3.232) and major bleeding (*HR* 4.126, 95%*CI* 1.022–16.664; *HR* 6.142, 95%*CI* 1.6789–22.369) compared with low morbidity group. In VKA subgroup, only high morbidity group was associated with a higher risk of all-cause death (*HR* 2.521, 95%*CI* 1.482–2.929), but not significantly in other events. For polypharmacy category, there were no significant statistics among these endpoints. Coronary artery disease (CAD), hypertension, chronic obstructive pulmonary disease, and antiplatelet therapy were independent predictors for VKA non-use in whole cohort, and patients with multimorbidity. CAD and antiplatelet therapy were independent predictors for VKA non-use in patients with polypharmacy.

**Conclusion:**

Multimorbidity was associated with worse outcomes in Chinese ED AF patients. Polypharmacy showed no significant statistics among these outcomes. CAD and antiplatelet therapy were independent risk factors of VKA non-use in Chinese ED AF patients with multimorbidity or polypharmacy.

## Introduction

Atrial fibrillation (AF) is the most common arrhythmia, currently worldwide estimated prevalence of AF in adults is 2~4% (1), in China, the AF prevalence is 1.8% among adults (aged ≥45 years) (2). The increasing burden of comorbidities, including heart failure (HF), hypertension, coronary artery disease (CAD), diabetes mellitus (DM), chronic obstructive pulmonary disease (COPD) and obesity are important AF risk factors, as well as increasing age (3). Moreover, these comorbidities accompany AF, it is estimated that up to 50% of AF patients have two or more concomitant diseases, multimorbidity (presence ≥ two comorbidities) is common among patients with AF (4). These patients have poor survival and higher stroke and bleeding risks compare with AF patients without multimorbidity (5), which poses a significant burden to patients, families, physicians, and global healthcare systems (3). The number of comorbidities directly related to polypharmacy (concomitant use of ≥ 5 medications) (6), and it reported that polypharmacy prevalence in AF has ranged from 40 to 95% (7). The high prevalence of polypharmacy increases further complexity to the management of AF patients.

As we known, AF is an independent risk factor for morbidity, stroke, HF, and rehospitalization. This risk is likely strong associated with multiple comorbidities and polypharmacy (8). 2020 European Society of Cardiology (ESC) AF guideline proposed AF better care (ABC) approach (3), the C component of the ABC approach include management of concomitant diseases. Management of concomitant disease and medications complements stroke prevention, improves survival, also improve compliance (3). The human body is a whole, to further improve the structured management of AF patients, promote patient compliance and values, and finally improve patient outcomes, there should be an integrative concept in AF treatment and management. Nowadays, more and more evidences have showed that integrated management of AF patients can reduce mortality and morbidity associated with AF, together with the need to reduce major adverse events in AF patients beyond just the risk of stroke, major bleeding, have inspired new idea concerning a multifaceted management of AF patients (9).

There are few available real-world registry data on comorbidities, polypharmacy, and adverse clinical outcomes in Chinese emergency department (ED) patients with AF. Understanding the patterns of multimorbid and polypharmacy in Chinese AF patients and characterizing their impact on AF-related outcomes are, therefore, the key to improving the overall or integrative management and outcomes of these patients in China.

## Methods

### Patients Selection

Chinese ED AF registry(CEAFR)was an observational, prospective, and multicenter registry study, details of study protocols and patients recruitment have been described previously (10, 11). Briefly, CEAFR was designed to enroll patients with AF who presented to an ED from Nov 2008 to Oct 2011. 20 sites represent different levels of medical care (urban and rural, academic and nonacademic, general and specialized) in China participated in the registry. The central administrative office of the study is located at the Fuwai Hospital, Beijing. The study was performed following the principle of the declaration of Helsinki and approved by the Fuwai Hospital Ethics committee. All patients signed a written informed consent.

The CHA_2_DS_2_-VASc (12) (congestive heart failure, hypertension, age≥75years, diabetes mellitus, previous stroke/transient ischemic attack, vascular disease, age 65 to 74 years, and sex category) scores were calculated after the first diagnosis of AF during hospital admission. Patients were divided into 3 groups according to the number of comorbidities in our study as low morbidity group (0–1 comorbidities), moderate morbidity group (2 comorbidities), and high morbidity group (≥3 comorbidities). Multimorbidity was defined as ≥2 comorbidities at admission. These cutoffs were based on a similar prior analysis (7). And based on the number of concomitant medications, polypharmacy in our study was defined as ≥5 medications.

### Follow-Up and Clinical Outcomes

Follow-up outcomes information about all-cause death, cardiovascular (CV) death, stroke, non-central nervous system (CNS) embolism, major bleeding, and hospitalization were obtained. Follow-up was completed in November 2012; the mean follow-up period was 12-month. All clinical adverse events were confirmed by reviewing the medical records.

The primary outcome was all-cause death during a 12-month follow-up. Secondary outcomes were CV death, stroke, major bleeding, and hospitalization. Outcomes were determined by a committee blinded to the therapy patients based on standardized definitions.

### Statistical Analysis

All data were assessed by two experienced research statisticians. Data were presented as means ± standard deviation (SD) or standard error (SE) for continuous variables and counts and proportions for categorical variables. The student's *T*-tests were used to assess the difference between continuous variables and the Pearson's Chi-squared test for categorical variables. Three group comparisons were conducted using analysis of variance (ANOVA), respectively. Estimates for outcomes were made by the Kaplan Meier method, and differences were assessed by the log-rank test. Univariate and multivariate Logistic regression or Cox's proportional hazards model with clustered standard errors was used for the outcomes analysis. In multivariable analyses, well-known risk factors and variables with a *P*-value of 0.05 were then entered into a multivariable analysis. Subgroup analyses were used to evaluate the homogeneity of the association of morbidity groups with or without vitamin K antagonist (VKA, warfarin), rate control, and rhythm treatment in all-cause death and CV death, stroke and major bleeding.

All tests were two-sided. A probability value of <0.05 was considered statistically significant. The SPSS 26.0 (IBM Corporation, New York, NY, USA) software package was used for statistical analysis.

## Results

### Baseline Characteristics

A total of 2015 patients were enrolled in CEAFR by the end of December 2011. Baseline characteristics of our cohort are shown in Table 1 and Table S1. In our cohort, the average age was 68.46±13.28 years, and average CHA_2_DS_2_-VASc score was 3.47±2.04, 1104 (54.8%) were female. The proportion of our cohort according to number of comorbid diseases are shown in Figure S1. The proportion of the low morbidity groups was 33%, moderate morbidity group was 30.2%, and high morbidity group was36.8%. Patients in High morbidity group were older, had higher blood pressure, higher CHA_2_DS_2_-VASc score, and higher proportional polypharmacy (Table 1).

**Table 1:** Baseline characteristic of AF patients by morbidity category.

	**Low morbidity group (0–1) *N* = 665**	**Moderate morbidity group (2) *N* = 608**	**High morbidity group (≥3) *N* = 742**	***P*-value**
Age, years	64.7 ± 14.8	67.5 ± 12.7	72.6 ± 11.1	<0.001
Female *n*, (%)	355 (53.4)	343 (56.4)	406 (54.7)	0.554
BMI (Kg/m^2^)	23.5 ± 3.3	23.4 ± 3.6	23.8 ± 3.9	0.075
SBP (mmHg)	126.6 ± 20.9	130.5 ± 23.8	137.8 ± 23.7	<0.001
DBP (mmHg)	78.4 ± 12.4	79.2 ± 14.9	81.8 ± 16.2	<0.001
HR (bpm)	105.5 ± 30.9	101.4 ± 29.4	98.5 ± 27.4	<0.001
CHA_2_DS_2_-VASc score	2.1 ± 1.6	3.2 ± 1.6	4.9 ± 1.8	<0.001
CHA_2_DS_2_-VASc≥2 *n*, (%)	380 (57.1)	518 (85.2)	725 (97.7)	<0.001
Current Smoking *n*, (%)	117 (17.6)	137 (22.5)	179 (24.1)	0.009
Current Drinking *n*, (%)	51 (7.7)	24 (3.9)	36 (4.9)	0.009
Type of AF *n*, (%)				<0.001
Paroxysmal	295 (44.4)	164 (27.0)	159 (21.4)	
Persistent	182 (27.4)	134 (22.0)	133 (17.9)	
Permanent	188 (28.3)	310 (51.0)	450 (60.6)	
Comorbidities *n*, (%)				
Coronary artery disease	69 (10.4)	234 (38.5)	540 (72.8)	<0.001
Hypertension	198 (29.8)	322 (53.0)	598 (80.6)	<0.001
Heart failure	35 (5.3)	245 (40.3)	474 (63.9)	<0.001
Valvular heart disease	70 (10.5)	172 (28.3)	165 (22.2)	<0.001
Congenital heart disease	5 (0.8)	15 (2.5)	23 (3.1)	0.008
Diabetes mellitus	15 (2.3)	48 (7.9)	248 (33.4)	<0.001
Previous stroke or TIA	15 (2.3)	79 (13.0)	285 (38.4)	<0.001
Previous major bleeding	4 (0.6)	10 (1.6)	34 (4.6)	<0.001
COPD	18 (2.7)	52 (8.6)	166 (22.4)	<0.001
Dementia or cognitive defects	0 (0.0)	2 (0.3)	42 (5.7)	<0.001
Sleep apnea	6 (0.9)	18 (3.0)	46 (6.2)	<0.001
Hyperthyroidism	20 (3.0)	19 (3.1)	27 (3.6)	0.777
Medications *n*, (%)				
Polypharmacy (≥5 medications)	84 (12.6)	174 (28.6)	312 (42.0)	<0.001
OAC				
Warfarin	121 (18.2)	138 (22.7)	116 (15.6)	0.004
TTR%^*^375 (±SE)	27.7 ± 2.6	29.1 ± 2.3	27.7 ± 2.8	0.903
TTR≥70%^*^375	12 (9.9)	11 (8.0)	12 (10.3)	0.782
Antiplatelet	333 (50.1)	400 (65.8)	548 (73.9)	<0.001
Aspirin	327 (49.2)	386 (63.5)	531 (71.6)	<0.001
Clopidogrel	30 (4.5)	49 (8.1)	83 (11.2)	<0.001
Rate control	405 (60.9)	504 (82.9)	624 (84.1)	<0.001
Beta-Blocker	307 (46.2)	311 (51.2)	397 (53.5)	0.021
CCB	126 (18.9)	172 (28.3)	275 (37.1)	<0.001
Digoxin	133 (20.0)	255 (41.9)	330 (44.5)	<0.001
Rhythm control	131 (19.7)	88 (14.5)	107 (14.4)	0.011
Amiodarone	98 (14.7)	69 (11.3)	80 (10.8)	0.056
Propafenone	35 (5.3)	22 (3.6)	33 (4.4)	0.365
Sotalol	5 (0.8)	4 (0.7)	4 (0.5)	0.883
Others				
Diuretic	141 (21.2)	281 (46.2)	435 (58.6)	<0.001
ARB	69 (10.4)	105 (17.3)	196 (26.4)	<0.001
ACEI	104 (15.6)	183 (30.1)	246 (33.2)	<0.001
Statins	89 (13.4)	154 (25.3)	281 (37.9)	<0.001

*AF, atrial fibrillation; BMI, body mass index; SBP, systolic blood pressure; DBP, diastolic blood pressure; HR, heart rate; CHA_2_DS_2_-VASc=congestive heart failure, hypertension, age≥75years, diabetes mellitus, previous stroke/transient ischemic attack, vascular disease, age 65 to 74 years, and sex category; TIA, transient ischemic attack; COPD, chronic obstructive pulmonary disease; OAC, oral anticoagulation; TTR, time in therapeutic range; SE, standard error; CCB, calcium channel blockers; ARB, angiotensin receptor blocker; ACEI, angiotensin-converting enzyme inhibitor*.

* *Data available only for patients on warfarin*.

Five hundred and seventy (28.3%) patients were polypharmacy in our cohort. The proportion of our cohort according to number of medications are shown in Figure S2. Compare to patients without polypharmacy, patients with polypharmacy also have higher blood pressure, higher CHA_2_DS_2_-VASc score, a higher number of co-morbidities, and more often CAD, hypertension, HF and DM (Table S1).

### Baseline Medications

Only 375 (18.6%) patients received VKA (warfarin) treatment, mean time in therapeutic range (TTR) was 28.2±1.5, and proportional of TTR≥70% was only 9.3%. As shown in Table 1, oral anticoagulation (OAC) use was more frequently used in patients with low and moderate morbidity groups, whereas antiplatelet strategy was more commonly used in high morbidity group. The rate control strategy was more commonly used in high morbidity group, whereas the rhythm control strategy was more frequently used in low morbidity group. The proportional of angiotensin converting enzyme inhibitor (ACEI), angiotensin receptor blocker (ARB), diuretics and statins usage were higher in patients with high morbidity group.

OAC, antiplatelet strategy, rate control strategy, rhythm control strategy, diuretic, ACEI, ARB and statins were more frequently used in patients with polypharmacy than patients without polypharmacy (all *p* < 0.05), but the TTR is higher in patients without polypharmacy compare with patients with polypharmacy (see Table S1).

### 12-Month Outcomes

**Figure 1:**
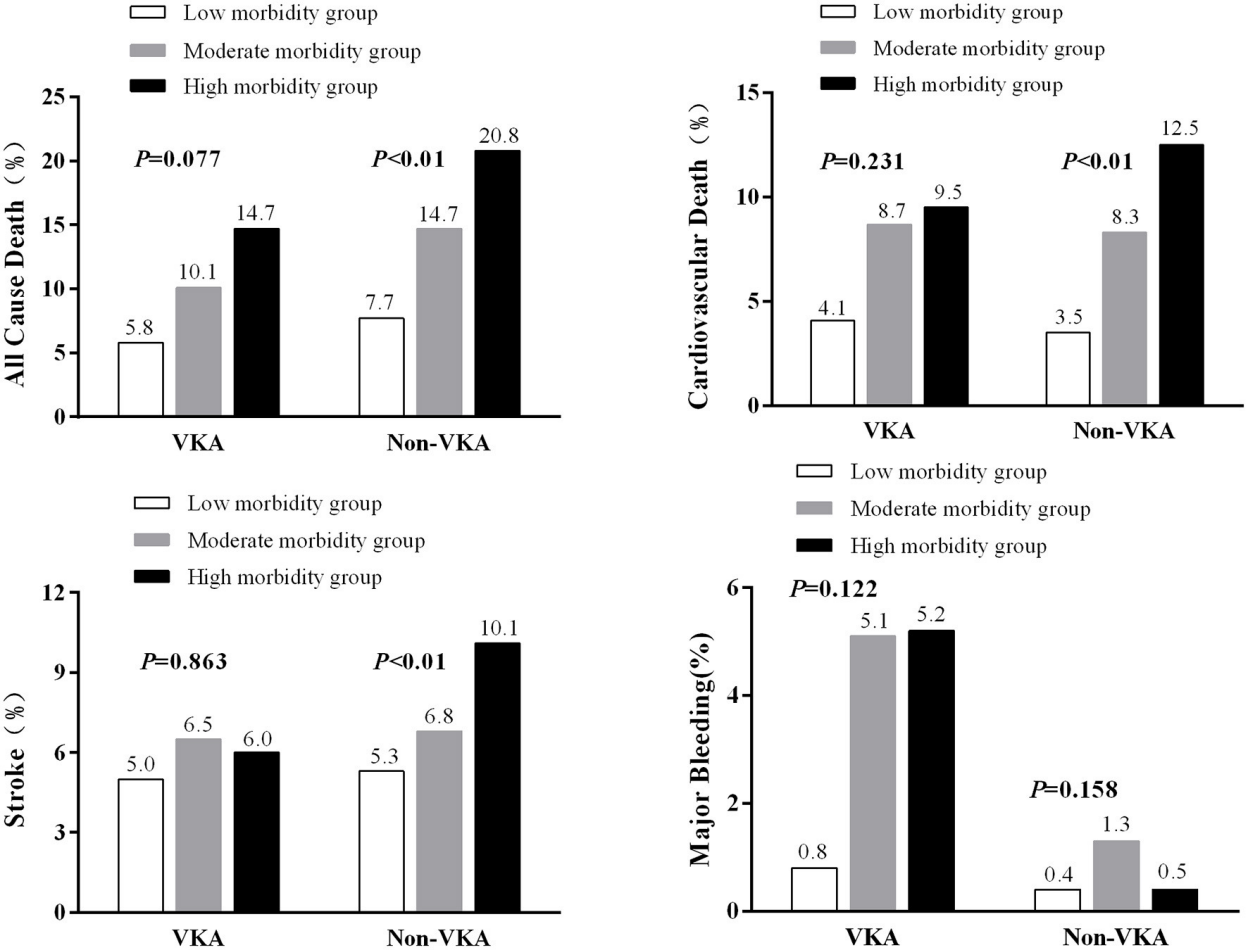
Event rates of outcome variables in patients with AF by morbidity categories and with or without VKA treatment.

Table 2 showed 12-month outcomes for all pre-defined endpoints in 3 morbidity groups. During 12-month follow-up, a total of 279 (13.8%) all-cause deaths and 164 (8.1%) CV deaths occurred. All-cause mortality, CV mortality, stroke and hospitalization were highest in high morbidity group patients. Moreover, the result remains significantly in patients without VKA treatment (Figure 1).

**Table 2:** Outcome event rates at 12 months in morbidity categories in follow-up.

	**Low morbidity group (0–1)**	**Moderate morbidity group (2)**	**High morbidity group (≥3)**	***P*-value**
All-cause death, *n* (%)	49 (7.4)	83 (13.7)	147 (19.8)	<0.001
Cardiovascular death, *n* (%)	24 (3.6)	51 (8.4)	89 (12.0)	<0.001
Stroke, *n* (%)	35 (5.3)	41 (6.7)	70 (9.4)	0.009
Non-CNS systemic embolism, *n* (%)	2 (0.3)	4 (0.7)	9 (1.2)	0.133
Major bleeding, *n* (%)	3 (0.5)	13 (2.1)	9 (1.2)	0.025
Hospitalization	158 (23.8)	193 (31.7)	272 (36.7)	<0.001
AF complications	129 (19.4)	124 (20.4)	148 (19.9)	0.905
Heart failure	43 (6.5)	99 (16.3)	183 (24.7)	<0.001
Myocardial infarction	2 (0.3)	4 (0.7)	16 (2.2)	0.327

*AF, atrial fibrillation; CNS, central nervous system*.

For morbidity groups, we compare VKA or Non-VKA treatment, Rhythm or Non-Rhythm treatment, Rate or Non-Rate treatment, in each morbidity group (Figures S5–S7). Although the events rate of all-cause death, cardiovascular death and stroke were higher in Non-VKA group, but there were no statistic difference. However, for major bleeding, the event rate was much higher in VKA treatment group, especially in moderate and high morbidity groups, and also showed significantly different. The events rates are lower in rhythm control group, especially in high morbidity group, although these did not reach the statistically significant. For rate control, only in low morbidity group, the all-cause mortality was lower in rate-control strategy, but we cannot see any trend in rate control for endpoints.

Outcomes at 12-month for polypharmacy groups are shown in Table S2,but in polypharmacy category, except hospitalization, other endpoints events were not significantly different.

### Survival and Regression Analysis

**Figure 2:**
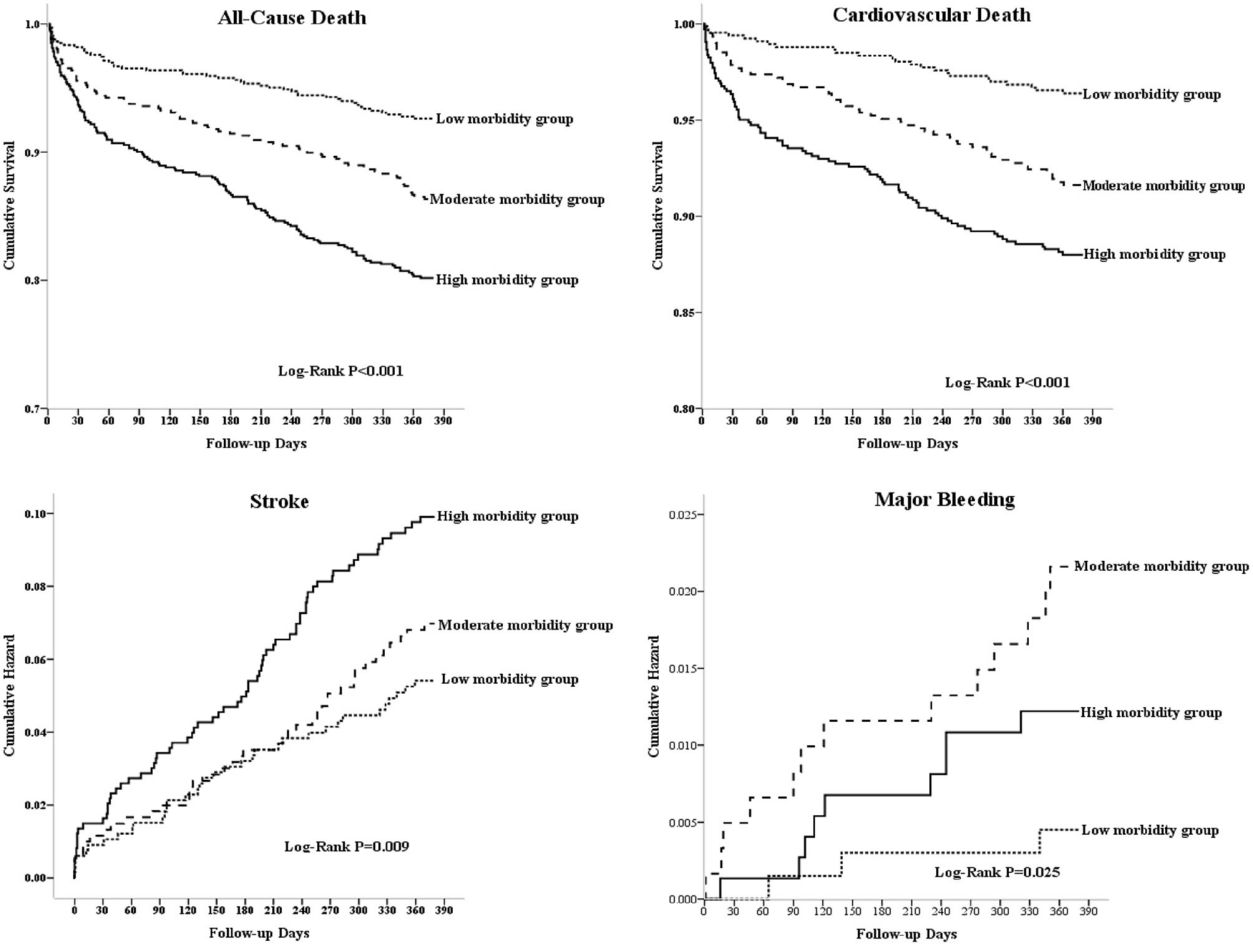
The Kaplan-Meyer survival curves and event rates for all-cause death, cardiovascular death, stroke, and major bleeding in patients with AF according to the morbidity categories.

Kaplan-Meier analysis of outcomes across different morbidity groups shows that risk for all-cause death, CV death, major bleeding and stroke were consistently the highest in the high morbidity group among the 3 groups (Figure 2). For polypharmacy category, there were no significantly statistics among these endpoints (Figure S3). Cause the proportion of patients treated with OAC was very low, and the TTR also very low, the survival for all-cause death was higher in the VKA usage group than Non-VKA group, and there are no difference in cardiovascular death and stroke. However, for major bleeding, the risk of major bleeding was higher in VKA group (Figure S4).

**Figure 3:**
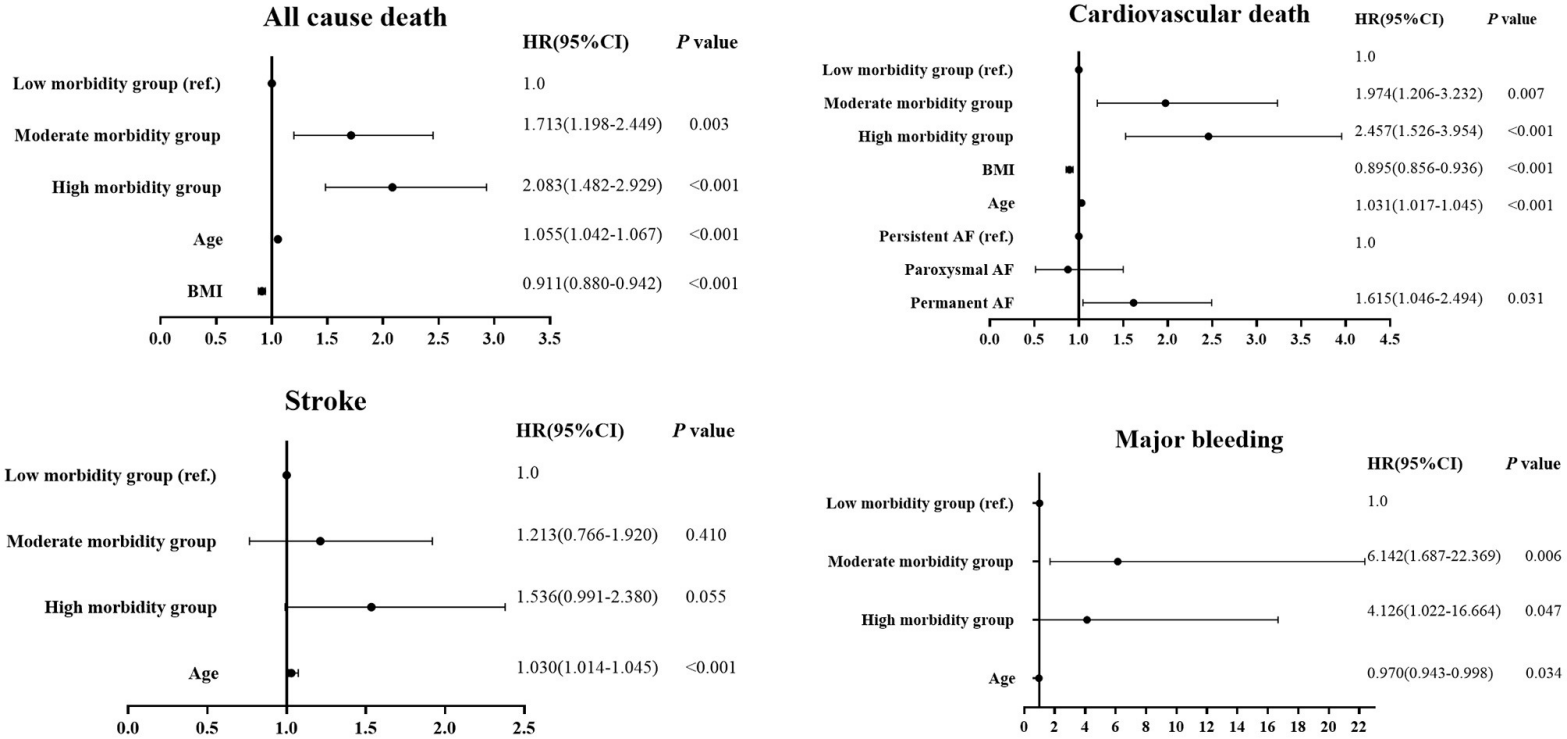
Multivariate Cox proportional models of predictors for all-cause death, cardiovascular death, stroke, and major bleeding by morbidity categories.

Final multivariate Cox proportional models of predictors for all-cause death, CV death, stroke and major bleeding were shown in Figure 3. Both high morbidity group and moderate morbidity group were significantly associated with a higher risk of all-cause death (hazard ratio (*HR)* 2.083, 95% confidence interval (*CI*)1.482–2.929, *p* < 0.001; *HR* 1.713, 95%*CI* 1.198–2.449, *p* = 0.003) and CV death (*HR* 2.457, 95%*CI* 1.526–3.954, *P* < 0.001; *HR* 1.974, 95%*CI* 1.206–3.232, *p* = 0.007) and major bleeding (*HR* 4.126, 95%*CI* 1.022–16.664, *p* = 0.047; *HR* 6.142, 95%*CI* 1.6789–22.369, *p* = 0.006) compared with low morbidity group. Table S3 shows the final multivariate logistic regression analysis for predictors of hospitalization. After adjustment for multiple relevant co-variables, moderate morbidity (odds ratio (*OR*) 1.357, 95%*CI* 1.036–1.776), high morbidity (*OR* 1.621, 95%*CI* 1.228–2.141) and polypharmacy (*OR* 1.527, 95%*CI* 1.229–1.897) were predictors of hospitalization.

**Figure 4:**
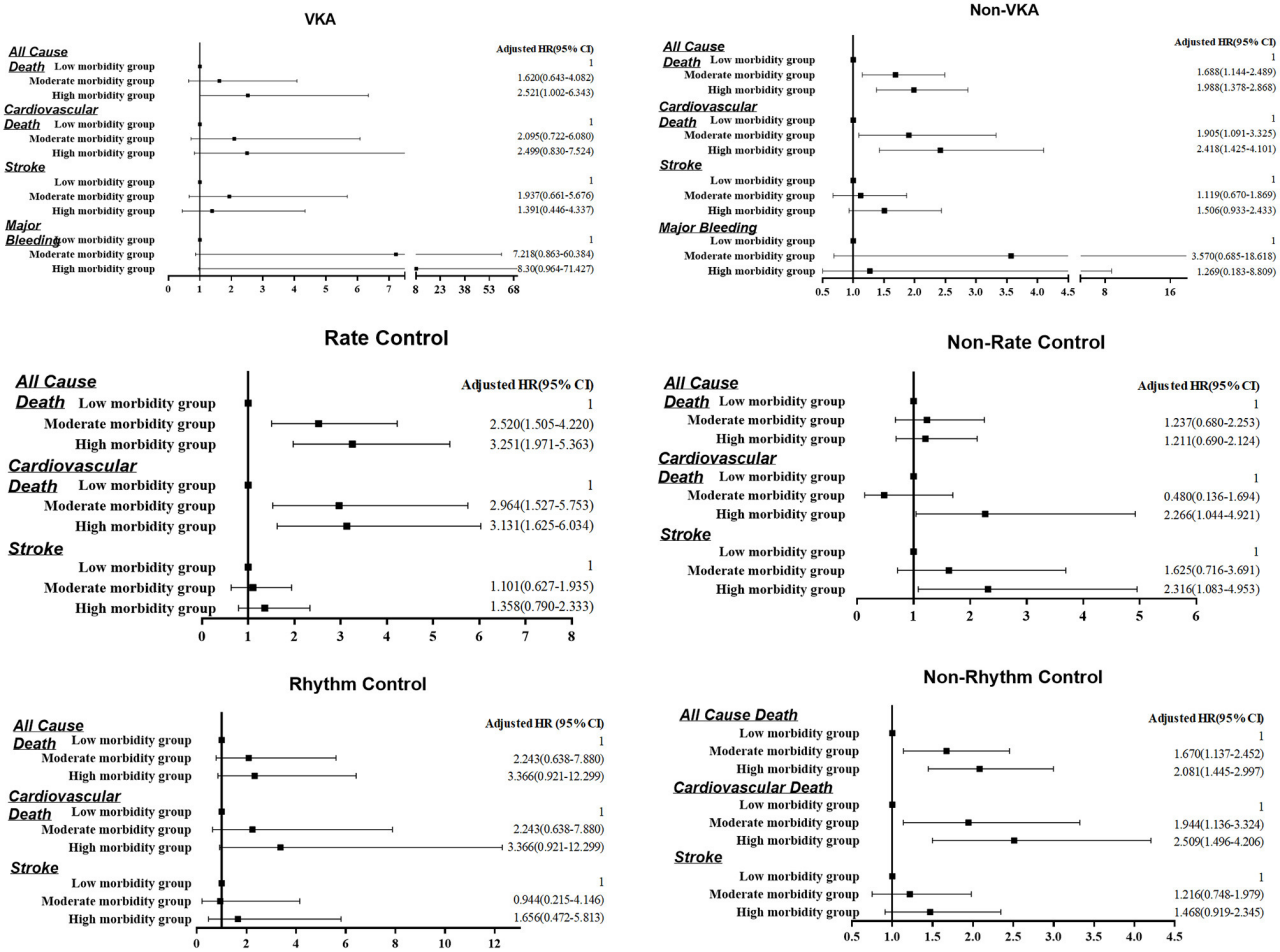
Multivariate Cox proportional models of predictors for all-cause death, cardiovascular death, stroke, and major bleeding by morbidity categories and different treatment strategies.

Figure 4 shows the subgroup analysis for different morbidity groups and outcomes in the condition of VKA treatment or not, rate control treatment or not, and rhythm control or not. In VKA treatment subgroup, only high morbidity group was associated with a higher risk of all-cause death [*HR* 2.521, 95%*CI* 1.482–2.929, *P* < 0.001)], but not significantly in other events. In non-VKA treatment, the trend of morbidity group associated with all-cause death and CV death remains exist. In the rate control treatment subgroup, both high morbidity and moderate morbidity groups were significantly associated with a higher risk of all-cause death and CV death. In non-rate control subgroup, all these events did not significantly different. In rhythm control subgroup, there are not statistically significant in all the endpoints events among morbidity group. Nevertheless, in non-rhythm control subgroup, both high and moderate morbidity groups were significantly associated with a higher risk of all-cause death and CV death.

### Predictors of OAC Non-use in Patients With Multimorbidity or Polypharmacy

The final multivariate Cox proportional models of predictors for OAC non-use were shown in Table 3. CAD, hypertension, COPD, and antiplatelet therapy were independent predictors of OAC non-use in the whole cohort and patients with multimorbidity. CAD and antiplatelet therapy were independent risk factors of OAC non-use in patients with polypharmacy.

**Table 3:** Independent predictors of OAC non-use in whole AF patients, in patients with multimorbidity or polypharmacy.

	**OR (95%CI)**	***P*-value**
Whole patients		
Hypertension	1.818 (1.409–2.346)	<0.001
CAD	1.493 (1.143–1.951)	0.003
COPD	1.633 (1.1190–2.404)	0.013
Antiplatelet	3.307 (2.573–4.251)	<0.001
Rate control	0.541 (0.402–0.729)	<0.001
Rhythm control	0.495 (0.373–0.657)	<0.001
Statins	0.607 (0.451–0.817)	0.001
ACEI	0.690 (0.526–0.906)	0.007
OAC non-use in AF patients with multimorbidity		
Hypertension	2.214 (1.597–3.069)	<0.001
CAD	1.853 (1.359–2.527)	<0.001
COPD	2.051 (1.348–3.119)	0.001
Antiplatelet	4.814 (3.519–6.586)	<0.001
Rhythm control	0.680 (0.467–0.989)	0.044
Statins	0.489 (0.345–0.694)	<0.001
ACEI	0.686 (0.500–0.940)	0.019
OAC non-use in AF patients with polypharmacy		
CAD	2.168 (1.417–3.317)	<0.001
Antiplatelet	7.090 (3.510–14.322)	<0.001

*OR, odds ratio; CI, confidence interval; AF, atrial fibrillation; OAC, oral anticoagulation; CAD coronary artery diseases; COPD, chronic obstructive pulmonary disease; ACEI, angiotensin-converting enzyme inhibitor*.

## Discussion

This study provides prospective multicenter data on multimorbidity and polypharmacy patterns among Chinese ED patients with AF. We found several results. First, Multimorbidity and polypharmacy are very common in our population. Second, moderate morbidity, high morbidity and polypharmacy were predictors of hospitalization. Third, CAD and antiplatelet therapy were independent risk factors of OAC non-use in AF patients with multimorbidity or polypharmacy. Forth, no association was found between polypharmacy and death, stroke and major bleeding in our study. Lastly, and most importantly, moderate and high morbidity groups were associated with the prespecified outcomes of all-cause death and cardiovascular death and consistent in non-VAK treatment and non-rhythm control subgroup.

Multimorbidity and polypharmacy were common among AF patients, multimorbidity was reported 69.5~80.4% prevalence (13, 14), and polypharmacy prevalence in AF has ranged from 40 to 95% according to study setting and population. The proportion of multimorbidity and polypharmacy in our study is 67% and 28.3%, respectively. For the presence of multimorbidity, both observational and randomized controlled trial have shown that the risk for all major adverse events related to AF patients is increased, especially all-cause mortality (5, 7). In our study, Multimorbidity also associated with increased risk of mortality. People with moderate and high morbidity groups have poorer health outcomes and higher mortality and major bleeding risk than people with low morbidity. Multimorbidity is a driver of polypharmacy. In our analysis, the proportion of polypharmacy in the high morbidity group was higher than moderate and low morbidity group, and the high morbidity group was on more antiplatelet therapy than those with low morbidity group. Similarly, the risk of CV events and death in AF patients with polypharmacy was significantly increased, and polypharmacy was associated with all-cause mortality, clinically relevant bleeding, and hospitalization in AF patients (6, 15, 16). In all these cases, an increased events rate, and increased correlation with risk of events found to be independent of other clinical features (17). Piccini et al. (6) showed increasing medication use was associated with higher risk of bleeding but not stroke in ROCKET AF. In our cohort, polypharmacy was associated with a higher hospitalization rate and also a predictor for hospitalization, but polypharmacy was not associated with mortality, stroke and major bleeding risk.

Nowadays, the “ABC” approach was proposed as a possible model to integrate the various main aspects related to AF management and streamline and facilitate the integrated care and the holistic evaluation of these patients (3). The ABC approach has also been shown to be associated with a lower risk of major adverse events in AF patients who have multiple comorbidities, use of polypharmacy (9); A recent meta-analysis have indeed showed that the prevalence of several comorbidities was a significant moderator of the effect of the ABC pathway on all-cause and CV death, and that the effect was different in magnitude across different CHA_2_DS_2_-VASc groups (which actually are related to the multimorbidity status) (18). These evidences would suggest that such a holistic approach is even more needed in those “clinically complex” AF patients.

For “A,” Anticoagulation/Avoid stroke is the most important and the first goal for AF patients' management. Patients often with multiple comorbidities remain at risk for serious adverse events without effective OAC (19). Proietti et al. (20) showed increased burden of multimorbidity is inversely associated with OAC prescription in patients with AF, which could significantly affect the clinical history of patients with AF, despite not influencing treatment discontinuation. Rasmussen et al. (21) showed multimorbidity was associated with lower odds of receiving OACs and rhythm control procedures in older Danish AF patients (age > 70 years). This presents a clinical conundrum as multimorbidity patients potentially benefit the most from treatment with OACs. An observational Spanish nationwide study (22) on anticoagulated AF patients showed multimorbidity was inversely associated with good anticoagulation control in AF anticoagulated patients. In our cohort, VKA therapy was the only OAC, also showed high morbidity patients had lower proportional VKA treatment. In the non-VAK subgroup, the trend of morbidity group associated with all-cause death and CV death existed, but in the VKA subgroup, these trends no longer remain significant.

The reason for nonprescription of OACs in multi-comorbidities AF patients, can associated with several factors including a history of hemorrhage and falling, cognitive impairment, frailty or physician concerns about interactions with concomitant medications (21, 23). In our study, in AF patients with multimorbidity or polypharmacy, the most common reason for non-prescription of VKA was CAD and antiplatelet treatment, this was similar with another United States-based ambulatory AF registry study (24), which examined non-valvular AF at elevated stroke risk (CHA_2_DS_2_-VASc ≥ 2) between January 5, 2008 and December 31, 2014, and find out antiplatelet use was prevalent and associated with the highest likelihood of OAC non-prescription. It worth discussing that, COPD was an important risk factor for VKA non-use in whole cohort and also in patients with multimorbidity. But a recent large meta-analysis (25), including 46 studies and with a total of 4232784 AF patients for quantitative synthesis, which showed COPD did not show significant difference for OAC prescription. In our cohort, COPD was associated with older age, higher proportional of CAD and HF, which may also affect the VKA prescription. We speculate that the presence of COPD indicates increased clinical complexity of AF patient management, and a perceived higher risk of major bleeding, resulting a lower OAC under-prescription.

However, the perception of the hazards of disease, unable to monitor regularly, afraid of bleeding, and economic factors, like universal medical insurance coverage, government medical expenditure, and dependence on individual payment vary greatly, these had affected the anticoagulation therapy in real-world AF patients in China. VKAs usage was limited by the narrow therapeutic interval, necessitating frequent INR monitoring and dose adjustments and many factors (such as concomitant drugs, genetics, etc.) would influence the effective of VKA anticoagulant. In our study, the major bleeding rate was much higher in VKA treatment group, especially in moderate and high morbidity groups, and also showed significantly different. These might because of the labile INR and low TTR, especially in multimorbidity AF patients, increasing risk of bleeding. With application of Non-vitamin K antagonist oral anticoagulant (NOAC), Piccini et al. (6) compared the adjustment results between rivaroxaban and warfarin based on the number of concomitant baseline medications in ROCKET AF, and showed rivaroxaban was tolerable in complex patients with multiple medications. Alexander et al. (26) showed the association between multimorbidity and clinical endpoints with the safety and effectiveness of apixaban and warfarin in a *post-hoc* ancillary analysis of the ARISTOTLE trial, and found that compared with warfarin, apixaban-treated patients with high multimorbidity (≥6 chronic conditions) had a better benefit, which may result in a reduction in bleeding and related healthcare expenditures.

For “B” —better symptom control, the second goal of AF patients management, is achieved by using rate or rhythm control. In our analysis, in rate control subgroup, both high and moderate morbidity groups were significantly associated with a higher risk of all-cause death and CV death. In rhythm control subgroup, there are not statistically significant in all the endpoints events among morbidity groups. Nevertheless, in the non-rhythm control subgroup, both high and moderate morbidity groups were significantly associated with a higher risk of all-cause death and CV death. Although these results may be confusing, they underscore the role that multimorbidity may affect the outcomes of rhythm control manage. Recent a prespecified EAST-AFNET 4 analysis compared the effect of early rhythm control therapy in asymptomatic patients (EHRA score I) to symptomatic patients, showed no difference in effectiveness of rhythm control strategy among symptomatic and asymptomatic patients, support the systematic, early initiation of rhythm control therapy in all patients with AF and concomitant cardiovascular conditions in dependent of their AF-related symptoms (27). Another prespecified sub-analysis of the randomized EAST-AFNET4 trial, find out rhythm control therapy conveys clinical benefit when initiated within 1 year of diagnosing AF in patients with signs or symptoms of HF (28). These perhaps speculative that an attempt to a rhythm control strategy should be pursued also in multimorbid AF patients. The high prevalence of multimorbidity in patients with AF and the influence of multimorbidity on the efficacy of AF therapy emphasizes the necessity of consider multimorbidity status when managing these patients. However, the net pros and cons of any AF treatment may change due to coexisting diseases and their concomitant therapies. Wherefore, assessing the interaction between AF treatments and multi-comorbidities is necessary to improve AF outcomes.

The “C” component of the ABC approach includes identifying and managing concomitant diseases. Our data replicated the finding that moderate and high morbidity were associated with worse outcomes in death, stroke, and major bleeding. Pharmacotherapy is the cornerstone of AF management, and combination treatments are common in AF patient with multimorbidity. A balance needs to be struck between the potential benefit of combining evidence-based therapies and the risk of adverse health outcomes. Managing AF through the ABC consistent approach is related to reducing major adverse events in clinically complex AF patients, including those with multimorbidity, polypharmacy, and hospitalization (9). This new approach and advocate the need for structured integrated, and holistic management of AF. Adding a routine evaluation of the burden of multimorbidity in patients' clinical evaluation could help identify patients who would benefit more from integrated and holistic management, which could reduce the overall risk of major adverse events beyond the baseline thromboembolic and bleeding risk (9).

In view of the rapid changes in lifestyles and demographic structure of the Chinese people, our findings of a high prevalence of multimorbidity or polypharmacy in Chinese ED patients with AF, will arouse attention to our country's health policies and practices. Chinese AF Patients with multimorbidity or polypharmacy are also consumers of healthcare resources and contribute to healthcare costs, preventable events in this population can save costs. Worth mentioning that, recently Guo and their team conducted mobile AF Application (mAFA)-II Trial (29), which was actually conducted in Chinese AF patients, high-risk patients with CHA_2_DS_2_-VASc score ≥3 in females and ≥2 in males using mAFA app had a high rate of OAC use (>80%), which demonstrated the efficacy of the ABC pathway in this specific population. This result convincing us to do more to integrated or holistic management approach in “clinical complex” AF patients. China urgently needs to pay attention to the national strategies for managing in Chinese AF patients with multimorbidity or polypharmacy.

## Limitations

Our study has some limitations to consider. First, this study is a retrospective analysis of prospectively collected data; however, we believe that the present real-world registry data reflects the status of AF management in China and has a clinical significance for the clinical management of AF patients with Multimorbidity and polypharmacy. Second, this study is a contemporary real-world population, and the AF cohort stopped recruiting in 2011 and NOACs were not approved for clinical use in China. Therefore, the present analysis did not clarify the effects of NOACs on the relationship between Multimorbidity, polypharmacy and adverse clinical events. Third, most of the concomitant diseases were cardiovascular diseases, this study did not consider an extensive list of chronic conditions, like kidney and liver diseases and infective diseases and soon, so it may under-reporting of some other system comorbidities. Lastly, the patients in the China ED AF Registry were only in selected institutions within a limited geographical area in China, and it is difficult to assume that our results can be generalized to all AF patients in China.

## Conclusion

In this 12-month follow-up analysis of a registry of “real world” Chinese patients with AF in ED, multimorbidity was associated with worse outcomes, moderate and high morbidity groups were associated with a higher risk of all-cause death and CV death, and consistent in non-VAK treatment and non-rhythm control subgroup. Polypharmacy was no a significantly statistic among these outcomes. CAD and antiplatelet therapy were independent risk factors of VKA non-use in patients with multimorbidity or polypharmacy. These findings emphasize the impact of multi chronic diseases and medications on the outcomes of AF and highlight the need to incorporate multimorbidity and polypharmacy management into the treatment guidelines for AF.

## Data Availability Statement

The raw data supporting the conclusions of this article will be made available by the authors, without undue reservation.

## Ethics Statement

The studies involving human participants were reviewed and approved by Ethics Committee of Fuwai Hospital. The patients/participants provided their written informed consent to participate in this study.

## Author Contributions

JW participated in literature search, study design, data collection, data analysis, data interpretation, and wrote the manuscript. HZ and X-hS. Shao carried out the data collection and analysis and provided the critical revision. Y-mY and JZ conceived of the study, participated in its design and coordination, and provided the critical revision. All authors have accepted responsibility for the entire content of this submitted manuscript and approved submission.

## Funding

This study was supported by research grants from the Capital's Funds for Research and Application of Clinical Diagnosis and Treatment Technology (No. Z191100006619121), National Center for Clinical Research in Cardiovascular Diseases (No. NCRC2020015), and Capital's Funds for Health Improvement and Research (No. 2018-2-4031).

## Conflict of Interest

The authors declare that the research was conducted in the absence of any commercial or financial relationships that could be construed as a potential conflict of interest.

## Publisher's Note

All claims expressed in this article are solely those of the authors and do not necessarily represent those of their affiliated organizations, or those of the publisher, the editors and the reviewers. Any product that may be evaluated in this article, or claim that may be made by its manufacturer, is not guaranteed or endorsed by the publisher.
