# Corrigendum: Multimorbidity and Polypharmacy in Chinese Emergency Department Patients With Atrial Fibrillation and Impacts on Clinical Outcomes

**DOI:** 10.3389/fcvm.2022.900118

**Published:** 2022-05-13

**Authors:** Juan Wang, Yan-min Yang, Jun Zhu, Han Zhang, Xing-hui Shao

**Affiliations:** Emergency and Intensive Care Center, State Key Laboratory of Cardiovascular Disease, Fuwai Hospital, National Center for Cardiovascular Disease, Chinese Academy of Medical Science and Peking Union Medical College, Beijing, China

**Keywords:** atrial fibrillation, multimorbidity, polypharmacy, vitamin K antagonist, outcomes, survival

There is an error in the Funding statement. The correct order for funding information is: **Capital's Funds for Research and Application of Clinical Diagnosis and Treatment Technology (No. Z191100006619121), National Center for Clinical Research in Cardiovascular Diseases (No. NCRC2020015), and Capital's Funds for Health Improvement and Research (No. 2018-2-4031)**.

The authors apologize for this error and state that this does not change the scientific conclusions of the article in any way. The original article has been updated.

## Publisher's Note

All claims expressed in this article are solely those of the authors and do not necessarily represent those of their affiliated organizations, or those of the publisher, the editors and the reviewers. Any product that may be evaluated in this article, or claim that may be made by its manufacturer, is not guaranteed or endorsed by the publisher.

