# Supplementary material for: Multimorbidity and Polypharmacy in Chinese Emergency Department Patients With Atrial Fibrillation and Impacts on Clinical Outcomes
Source: Front Cardiovasc Med. 2022 Jan 28;9:806234. doi: 10.3389/fcvm.2022.806234 (PMC8831736; doi:10.3389/fcvm.2022.806234)
Supplement: Supplementary file 1 [file Data_Sheet_1.docx]

***Supplementary material***

**Multimorbidity and polypharmacy in Chinese emergency department patients with atrial fibrillation and impacts on clinical outcomes**

Juan Wang, Yan-min Yang*, Jun Zhu, Han Zhang, Xing-hui Shao

Emergency and Intensive Care Center, State Key Laboratory of Cardiovascular Disease, Fuwai Hospital, National Center for Cardiovascular Disease, Chinese Academy of Medical Science and Peking Union Medical College, Beijing, 100037, China

*Corresponding author. Yan-min Yang, MD, Emergency and Intensive Care Center, State Key Laboratory of Cardiovascular Disease, Fuwai Hospital, National Center for Cardiovascular Disease, Chinese Academy of Medical Science and Peking Union Medical College, Beijing, 100037, China

Present address：No.167 Beilishi Road, Xicheng District, Beijing, 100037, China.

Email: [yymfuwai@163.com](mailto:yymfuwai@163.com) /[ymyang2014@163.com](mailto:ymyang2014@163.com)

Tel: 86-010-88322801; Fax: 86-010-88364591

| **Supplementary Table S1** Baseline characteristic of AF patients by polypharmacy category | | | |
| --- | --- | --- | --- |
|  | **With polypharmacy(≥5 medications)**  **N=570** | **without** **polypharmacy(＜5 medications)**  **N=1445** | ***P*-value** |
| Age, years | 68.8±11.3 | 68.3±14.0 | 0.466 |
| Female n,(%) | 313(54.9) | 791(54.7) | 0.944 |
| BMI(Kg/m^2^) | 24.0±3.8 | 23.4±3.5 | <0.001 |
| SBP(mmHg) | 136.0±23.8 | 130.3±22.9 | <0.001 |
| DBP(mmHg) | 82.8±15.2 | 78.8±14.4 | <0.001 |
| HR(bpm) | 100.3±28.2 | 102.2±29.8 | 0.193 |
| CHA_2_DS_2_-VASc score | 3.8±1.9 | 3.3±2.1 | <0.001 |
| Current Smoking n,(%) | 144(25.3) | 289(20.0) | 0.010 |
| Current Drinking n,(%) | 22(3.9) | 89(6.2) | 0.042 |
| **Type of AF n,**(%) |  |  | <0.001 |
| Paroxysmal | 154(27.0) | 464(32.1) |  |
| Persistent | 95(16.7) | 354(24.5) |  |
| Permanent | 321(56.3) | 627(43.4) |  |
| **Comorbidities n,**(%) |  |  |  |
| **Number of comorbidities** | 2.7±1.2 | 1.9±1.3 | <0.001 |
| Coronary artery disease | 341(59.8) | 502(34.7) | <0.001 |
| Hypertension | 404(70.9) | 714(49.4) | <0.001 |
| Heart Failure | 318(55.8) | 436(30.2) | <0.001 |
| Valvular heart disease | 128(22.5) | 279(19.3) | 0.113 |
| Congenital heart disease | 8(1.4) | 35(2.4) | 0.154 |
| Diabetes mellitus | 114(20.0) | 197(13.6) | <0.001 |
| Previous Stroke or TIA | 113(19.8) | 266(18.4) | 0.464 |
| Previous major bleeding | 14(2.5) | 34(2.4) | 0.891 |
| COPD | 59(10.4) | 177(12.2) | 0.233 |
| Dementia or cognitive defects | 7(1.2) | 37(2.6) | 0.065 |
| Sleep apnea | 18(3.2) | 52(3.6) | 0.627 |
| Hyperthyroidism | 12(2.1) | 54(3.7) | 0.064 |
| **Medications n,(%)** |  |  |  |
| **OAC** |  |  |  |
| Warfarin | 144(25.3) | 231(16.0) | <0.001 |
| TTR%*375(±SE) | 22.6±2.3 | 31.7±1.9 | 0.003 |
| TTR≥70%*375 | 10(6.9) | 25(10.8) | 0.209 |
| **Antiplatelet** | 511(89.6) | 770(53.3) | <0.001 |
| Aspirin | 502(88.1) | 742(51.3) | <0.001 |
| Clopidogrel | 99(17.4) | 63(4.4) | <0.001 |
| **Rate control** | 557(97.7) | 976(67.5) | <0.001 |
| Beta-Blocker | 435(76.3) | 580(40.1) | <0.001 |
| CCB | 251(44.0) | 322(22.3) | <0.001 |
| Digoxin | 339(59.5) | 379(26.2) | <0.001 |
| **Rhythm control** | 157(27.5) | 169(11.7) | <0.001 |
| Amiodarone | 130(22.3) | 117(8.1) | <0.001 |
| Propafenone | 42(7.4) | 48(3.3) | <0.001 |
| Sotalol | 8(1.4) | 5(0.3) | 0.008 |
| **Other** |  |  |  |
| Diuretic | 423(74.2) | 434(30.0) | <0.001 |
| ARB | 193(33.9) | 177(12.2) | <0.001 |
| ACEI | 329(57.7) | 204(14.1) | <0.001 |
| Statins | 309(54.2) | 219(15.2) | <0.001 |

AF, atrial fibrillation; BMI, body mass index; SBP, systolic blood pressure; DBP, diastolic blood pressure; HR, heart rate; CHA_2_DS_2_-VASc=congestive heart failure, hypertension, age≥75years, diabetes mellitus, previous stroke/transient ischemic attack, vascular disease, age 65 to 74 years, and sex category; TIA, transient ischemic attack; COPD, chronic obstructive pulmonary disease; OAC, oral anticoagulation; TTR, time in therapeutic range; SE, standard error; CCB, calcium channel blockers; ARB, angiotensin receptor blocker; ACEI, angiotensin-converting enzyme inhibitor

*Data available only for patients on warfarin.

| **Supplementary Table S2** Outcome event rates at 12 months in polypharmacy categories in follow-up | | | |
| --- | --- | --- | --- |
|  | **with polypharmacy** | **Without polypharmacy** | ***P*-value** |
| **All-cause Death,n(%)** | 75(13.2) | 204(14.1) | 0.574 |
| **Cardiovascular death,n(%)** | 54(9.5) | 110(7.6) | 0.169 |
| **Stroke, n(%)** | 40(7.0) | 106(7.3) | 0.804 |
| Non-CNS systemic embolism,n(%) | 2(0.4) | 13(0.9) | 0.197 |
| **Major Bleeding,n(%)** | 7(1.2) | 18(1.2) | 0.974 |
| **Hospitalization** | 223(39.1) | 400(27.7) | <0.001 |
| AF Complications | 133(23.3) | 268(18.5) | 0.015 |
| Heart Failure | 146(25.6) | 179(12.4) | <0.001 |
| Myocardial Infarction | 13(2.3) | 9(0.6) | 0.001 |

AF, atrial fibrillation; CNS, central nervous system

| **Supplementary TableS3** Independent predictors of Hospitalization in AF patients | | |
| --- | --- | --- |
| variables | OR (95%CI) | P-value |
| SBP | 0.993(0.987-0.999) | 0.020 |
| Comorbidities |  |  |
| Low morbidity group | reference |  |
| **Moderate morbidity group** | 1.357(1.036-1.776) | 0.026 |
| **High morbidity group** | 1.621(1.228-2.141) | 0.001 |
| Medications |  |  |
| without polypharmacy | reference |  |
| **Polypharmacy** | 1.527(1.229-1.897) | <0.001 |

AF, atrial fibrillation; OR, odds ratio; CI, confidence interval

Models were adjusted for age, sex, heart rate, systolic blood pressure, diastolic blood pressure, BMI, type of atrial fibrillation, current smoking, current drinking, CHS_2_DS_2_-VASc score, morbidity categories and polypharmacy categories.

| **Table S4** Baseline characteristic of AF patients by age-adjusted Charlson Comorbidity Index^＆^ | | | |
| --- | --- | --- | --- |
|  | **CCI Score 0-3**  **N=630** | **CCI Score≥4**  **N=1385** | ***P*-value** |
| Age, years | 53.7±10.4 | 75.2±7.9 | <0.001 |
| Female n,(%) | 336(53.3) | 768(55.5) | 0.376 |
| BMI(Kg/m^2^) | 23.9±3.7 | 23.4±3.6 | 0.004 |
| SBP(mmHg) | 124.8±21.7 | 135.1±23.3 | <0.001 |
| DBP(mmHg) | 79.0±14.2 | 80.3±15.0 | 0.055 |
| HR(bpm) | 105.8±30.9 | 99.9±28.5 | <0.001 |
| CHA_2_DS_2_-VASc score | 1.4±1.0 | 4.4±1.7 | <0.001 |
| Current Smoking n,(%) | 143(22.7) | 290(20.9) | 0.373 |
| Current Drinking n,(%) | 56(8.9) | 55(4.0) | <0.001 |
| **Type of AF n,**(%) |  |  | <0.001 |
| Paroxysmal | 245(38.9) | 373(26.9) |  |
| Persistent | 155(24.6) | 294(21.2) |  |
| Permanent | 230(36.5) | 718(51.8) |  |
| **Comorbidities n,**(%) |  |  |  |
| Coronary artery disease | 102(16.2) | 741(53.5) | <0.001 |
| Myocardial infarction | 9(1.4) | 139(10.0) | <0.001 |
| Hypertension | 227(36.0) | 891(64.3) | <0.001 |
| Heart Failure | 155(24.6) | 599(43.2) | <0.001 |
| Valvular heart disease | 203(32.2) | 204(14.7) | <0.001 |
| Congenital heart disease | 24(3.8) | 19(1.4) | <0.001 |
| Diabetes mellitus | 26(4.1) | 285(20.6) | <0.001 |
| Previous Stroke or TIA | 25(4.0) | 354(25.6) | <0.001 |
| Previous major bleeding | 15(2.4) | 33(2.4) | 0.998 |
| COPD | 5(0.8) | 231(16.7) | <0.001 |
| Dementia or cognitive defects | 1(0.2) | 43(3.1) | <0.001 |
| Sleep apnea | 29(4.6) | 1.4(3.0) | 0.062 |
| Hyperthyroidism | 36(5.7) | 30(2.2) | <0.001 |
| **Medications n,(%)** |  |  |  |
| **OAC** |  |  |  |
| Warfarin | 167(26.5) | 205(15.0) | <0.001 |
| TTR%*375(±SE) | 30.5±2.1 | 26.3±2.1 | 0.163 |
| TTR≥70%*375 | 14(8.4) | 21(10.1) | 0.571 |
| **Antiplatelet** | 346(54.9) | 935(67.5) | <0.001 |
| Aspirin | 340(54.0) | 904(65.3) | <0.001 |
| Clopidogrel | 28(4.4) | 134(9.7) | <0.001 |
| **Rate control** | 459(72.9) | 1074(77.5) | 0.022 |
| Beta-Blocker | 330(53.4) | 685(49.5) | 0.224 |
| CCB | 120(19.0) | 453(32.7) | <0.001 |
| Digoxin | 224(35.6) | 494(35.7) | 0.961 |
| **Rhythm control** | 120(19.0) | 206(14.9) | 0.018 |
| Amiodarone | 85(13.5) | 162(11.7) | 0.255 |
| Propafenone | 39(6.2) | 51(3.7) | 0.012 |
| Sotalol | 7(1.1) | 6(0.4) | 0.078 |
| **Other** |  |  |  |
| Diuretic | 229(36.3) | 628(45.3) | <0.001 |
| ARB | 73(11.6) | 297(21.4) | <0.001 |
| ACEI | 141(22.4) | 392(28.3) | 0.005 |
| Statins | 97(15.4) | 431(31.1) | <0.001 |

AF, atrial fibrillation; CCI, Charlson Comorbidity Index; BMI, body mass index; SBP, systolic blood pressure; DBP, diastolic blood pressure; HR, heart rate; CHA_2_DS_2_-VASc=congestive heart failure, hypertension, age≥75years, diabetes mellitus, previous stroke/transient ischemic attack, vascular disease, age 65 to 74 years, and sex category; TIA, transient ischemic attack; COPD, chronic obstructive pulmonary disease; OAC, oral anticoagulation; TTR, time in therapeutic range; SE, standard error; CCB, calcium channel blockers; ARB, angiotensin receptor blocker; ACEI, angiotensin-converting enzyme inhibitor

*Data available only for patients on warfarin.

^＆^Including myocardial infarct, heart failure, cerebrovascular disease, dementia, chronic pulmonary disease, diabetes and age.

| **Supplementary Table S5** Outcome event rates at 12 months by age-adjusted Charlson Comorbidity Index^＆^ | | | |
| --- | --- | --- | --- |
|  | **CCI Score 0-3**  **N=630** | **CCI Score≥4**  **N=1385** | ***P*-value** |
| **All-cause Death, n(%)** | 26(4.1) | 253(18.3) | <0.001 |
| **Cardiovascular death, n(%)** | 19(3.0) | 145(10.5) | <0.001 |
| **Stroke, n(%)** | 21(3.3) | 125(9.0) | <0.001 |
| Non-CNS systemic embolism, n(%) | 4(0.6) | 11(0.8) | 0.700 |
| **Major Bleeding, n(%)** | 9(1.4) | 16(1.2) | 0.607 |
| **Hospitalization** | 163(25.9) | 460(33.2) | 0.001 |
| AF Complications | 126(20.0) | 275(19.9) | 0.940 |
| Heart Failure | 59(9.4) | 266(19.2) | <0.001 |
| Myocardial Infarction | 0(0.0) | 22(1.6) | 0.001 |

AF, atrial fibrillation; CCI, Charlson Comorbidity Index; CNS, central nervous system

^＆^Including myocardial infarct, heart failure, cerebrovascular disease, dementia, chronic pulmonary disease, diabetes and age.

| **Table S6** Baseline characteristic and outcomes of AF patients with or without COPD | | | |
| --- | --- | --- | --- |
|  | **With COPD**  **N=236** | **Without COPD**  **N=1779** | ***P*-value** |
| Age, years | 75.5±9.1 | 67.5±13.5 | **<0.001** |
| Female n,(%) | 116(49.2) | 988(55.5) | 0.064 |
| BMI(Kg/m^2^) | 23.1±3.8 | 23.6±3.6 | 0.889 |
| SBP(mmHg) | 138±24.9 | 131±23.0 | 0.374 |
| DBP(mmHg) | 81.6±16.1 | 79.7±14.5 | **0.015** |
| HR(bpm) | 103.1±27.5 | 101.5±29.6 | 0.129 |
| CHA_2_DS_2_-VASc score | 4.3±1.9 | 3.4±2.0 | **0.043** |
| Current Smoking n,(%) | 77(32.6) | 356(20.0) | **<0.001** |
| Current Drinking n,(%) | 8(3.4) | 103(5.8) | 0.129 |
| **Type of AF n,**(%) |  |  | **0.004** |
| Paroxysmal | 54(22.9) | 564(31.7) |  |
| Persistent | 48(20.3) | 401(22.5) |  |
| Permanent | 134(56.8) | 814(45.8) |  |
| **Comorbidities n,**(%) |  |  |  |
| Coronary artery disease | 131(55.5) | 712(40.0) | **<0.001** |
| Myocardial infarction | 21(8.9) | 127(7.1) | 0.330 |
| Hypertension | 138(58.5) | 980(55.1) | 0.325 |
| Heart Failure | 113(47.9) | 641(36.0) | **<0.001** |
| Congenital heart disease | 3(1.3) | 40(2.2) | 0.329 |
| Diabetes mellitus | 38(16.1) | 273(15.3) | 0.763 |
| Previous Stroke or TIA | 53(22.5) | 326(18.3) | 0.127 |
| Previous major bleeding | 7(3.0) | 41(2.3) | 0.531 |
| Dementia or cognitive defects | 9(3.8) | 35(2.0) | 0.068 |
| Sleep apnea | 8(3.4) | 62(3.5) | 0.940 |
| Hyperthyroidism | 5(2.1) | 61(3.4) | 0.288 |
| **Medications n,(%)** |  |  |  |
| **OAC** |  |  |  |
| Warfarin | 28(11.9) | 347(19.5) | **0.005** |
| TTR%*375(±SE) | 22.6±5.8 | 28.7±1.5 | 0.747 |
| TTR≥70%*375 | 2(7.1) | 33(9.5) | 0.679 |
| **Antiplatelet** | 154(65.3) | 1127(63.4) | 0.568 |
| **Rate control** | 173(73.3) | 1360(76.4) | 0.288 |
| **Rhythm control** | 27(11.4) | 299(16.8) | **0.035** |
| **Outcomes, n(%)** |  |  |  |
| **All-cause Death,** | 61(25.8) | 218(12.3) | **<0.001** |
| **Cardiovascular death** | 29(12.3) | 135(7.6) | **0.013** |
| **Stroke** | 18(7.6) | 128(7.2) | 0.810 |
| **Major Bleeding** | 2(0.8) | 23(1.3) | 0.561 |

AF, atrial fibrillation; BMI, body mass index; SBP, systolic blood pressure; DBP, diastolic blood pressure; HR, heart rate; CHA_2_DS_2_-VASc=congestive heart failure, hypertension, age≥75years, diabetes mellitus, previous stroke/transient ischemic attack, vascular disease, age 65 to 74 years, and sex category; TIA, transient ischemic attack; COPD, chronic obstructive pulmonary disease; OAC, oral anticoagulation; TTR, time in therapeutic range; SE, standard error; CCB, calcium channel blockers; ARB, angiotensin receptor blocker; ACEI, angiotensin-converting enzyme inhibitor

*Data available only for patients on warfarin.

| **Table S7 Univariable analysis for risk of outcomes in all AF patients** | | | | | | |
| --- | --- | --- | --- | --- | --- | --- |
|  | **All-cause Death** | | **Cardiovascular Death** | | **Stroke** | |
| Variable | **HR (95%CI)** | ***P*-value** | **HR (95%CI)** | ***P*-value** | **HR (95%CI)** | ***P*-value** |
| Co-morbidities |  |  |  |  |  |  |
| Coronary artery disease | 1.239(0.979-1.568) | 0.074 | 1.099(0.808-1.497) | 0.547 | 1.330(0.961-1.839) | 0.085 |
| Hypertension | 1.123(0.885-1.425) | 0.338 | 1.083(0.795-1.476) | 0.613 | 1.609(1.141-2.269) | **0.007** |
| **Heart Failure** | **1.801(1.424-2.277)** | **<0.001** | **3.121(2.268-4.293)** | **<0.001** | **0.735(0.518-1.045)** | **0.086** |
| Valvular heart disease | 0.939(0.697-1.263) | 0.676 | 1.381(0.973-1.961) | 0.071 | 0.694(0.441-1.092) | 0.114 |
| Congenital heart disease | 0.852(0.352-2.064) | 0.723 | 1.179(0.437-3.180) | 0.745 | 0.631(0.156-2.548) | 0.518 |
| Diabetes mellitus | 1.491(1.117-1.991) | **0.007** | 1.347(0.916-1.982) | 0.130 | 1.309(0.867-1.976) | 0.200 |
| **Previous Stroke or TIA** | **1.641(1.258-2.140)** | **<0.001** | **1.243(0.859-1.800)** | **0.248** | **2.267(1.609-3.197)** | **<0.001** |
| Previous major bleeding | 1.049(0.495-2.221) | 0.901 | 0.246(0.034-1.757) | 0.162 | 2.154(1.008-4.603) | **0.048** |
| **COPD** | **2.315(1.743-3.075)** | **<0.001** | **1.668(1.117-2.491)** | **0.012** | **1.077(0.658-1.764)** | **0.769** |
| Dementia or cognitive defects | 3.122(1.885-5.172) | **<0.001** | 2.142(1.004-4.566) | **0.049** | 3.607(1.897-6.855) | **<0.001** |
| Sleep apnea | 0.689(0.325-1.459) | 0.331 | 0.506(0.161-1.584) | 0.242 | 0.778(0.288-2.102) | 0.621 |
| Hyperthyroidism | 0.732(0.346-1.549) | 0.414 | 0.726(0.269-1.959) | 0.528 | 1.276(0.563-2.888) | 0.559 |

AF, atrial fibrillation; HR, hazard ratio; CI, confidence interval; TIA, transient ischemic attack; COPD, chronic obstructive pulmonary disease.


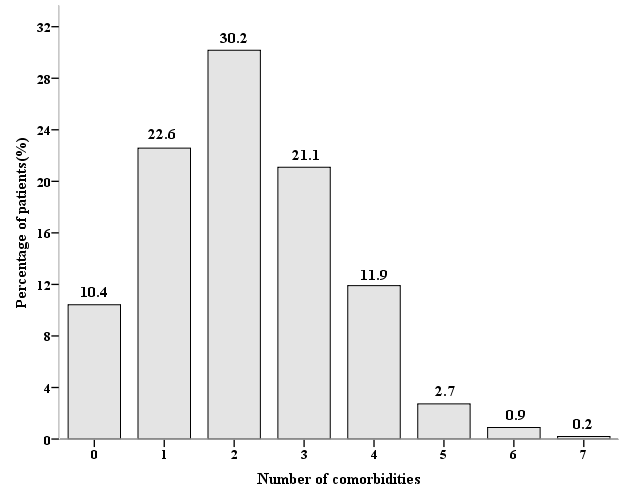


**Supplement Figure S1** Proportion of patients with AF according to number of comorbid diseases


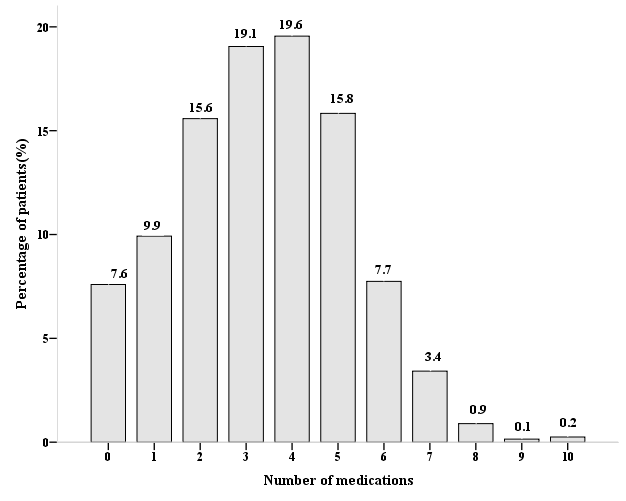


**Supplement Figure S2** Proportion of patients with AF according to number of medications


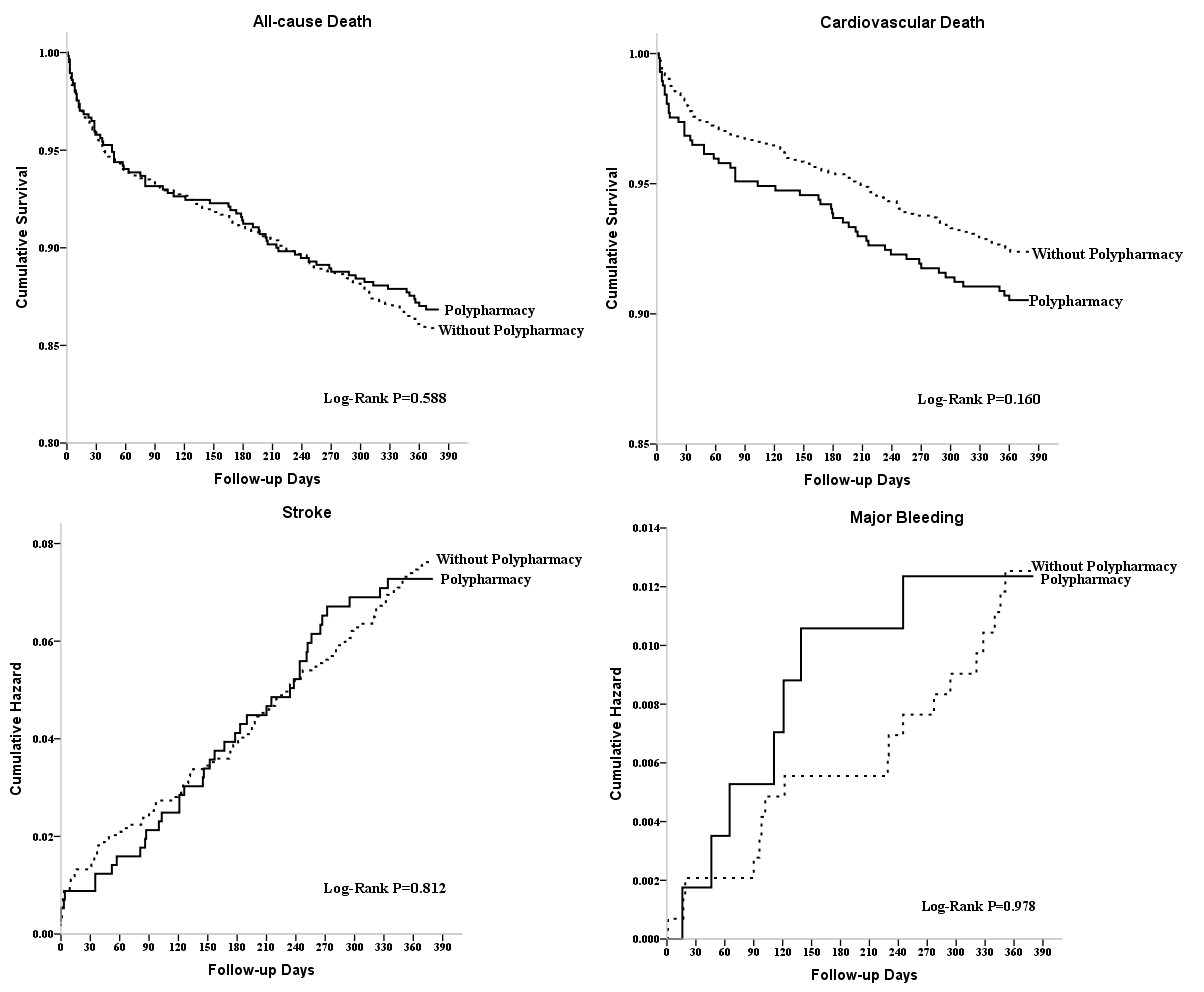


**Supplement Figure S3** The Kaplan-Meyer survival curves for all-cause death, cardiovascular death, stroke and major bleeding in patients with AF according to polypharmacy categories


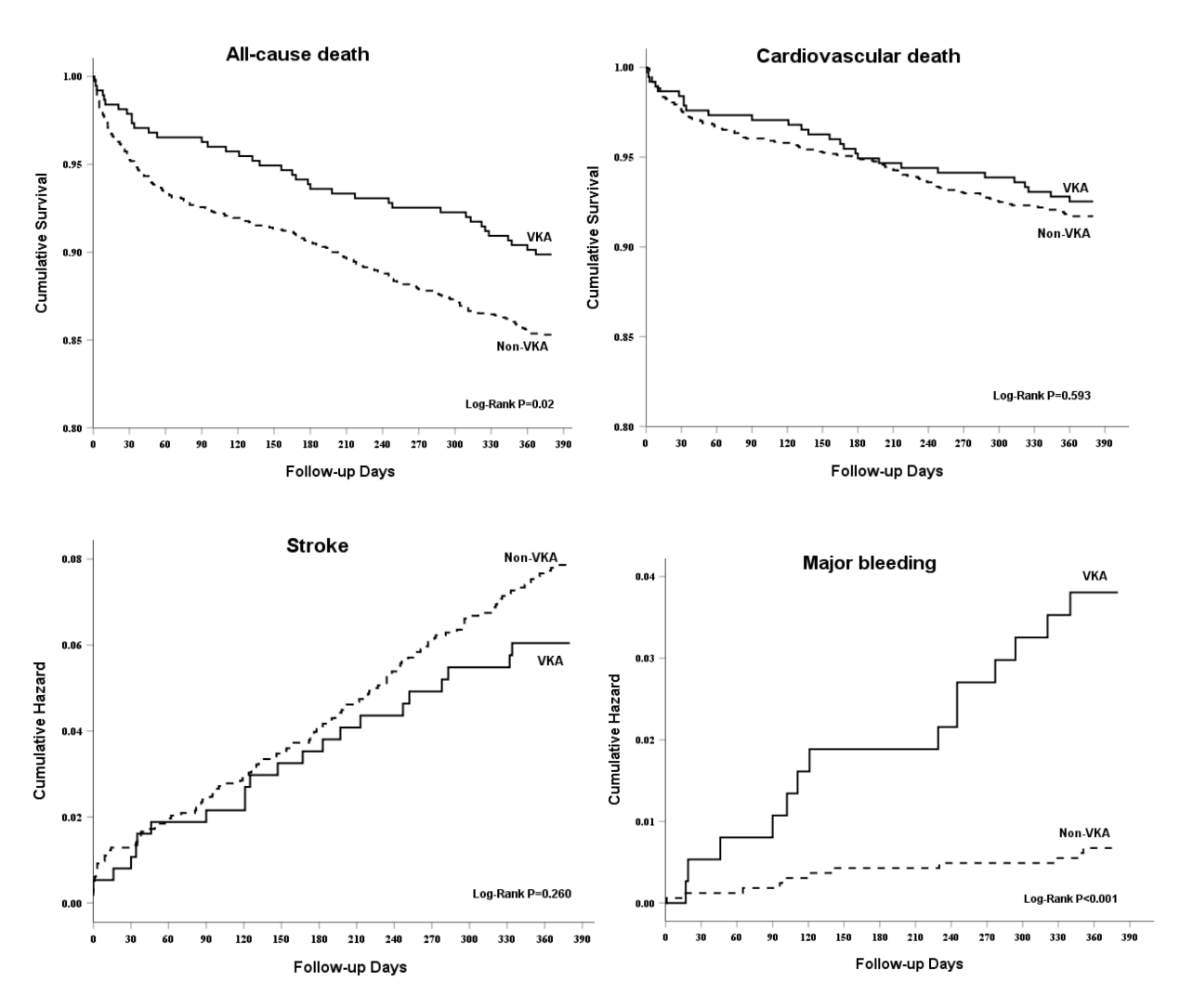


**Supplement Figure S4** The Kaplan-Meyer survival curves for all-cause death, cardiovascular death, stroke and major bleeding in patients with AF according to VKA or Non-VKA usage


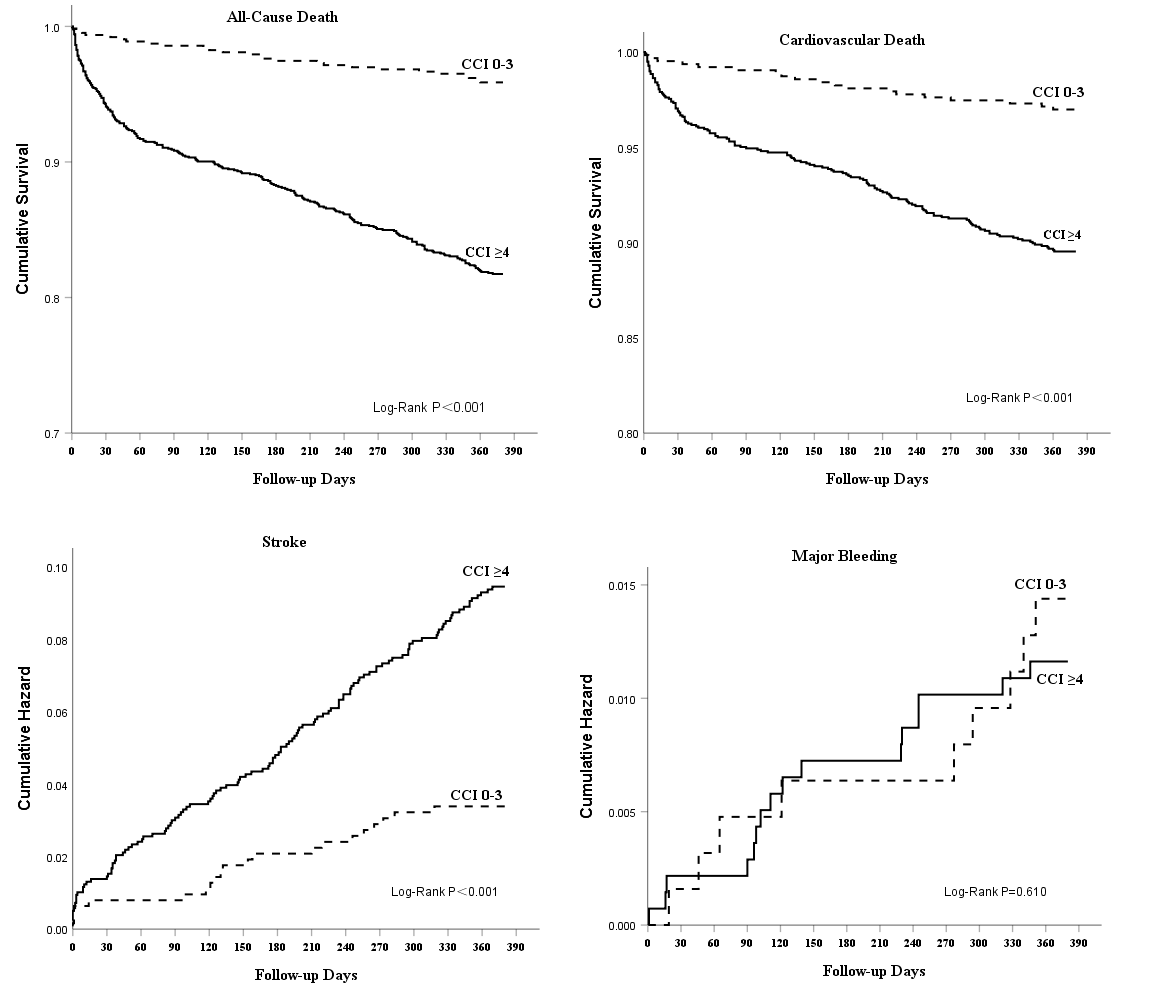


**Supplement Figure S5** The Kaplan-Meyer survival curves for all-cause death, cardiovascular death, stroke and major bleeding in patients with AF according to CCI score.


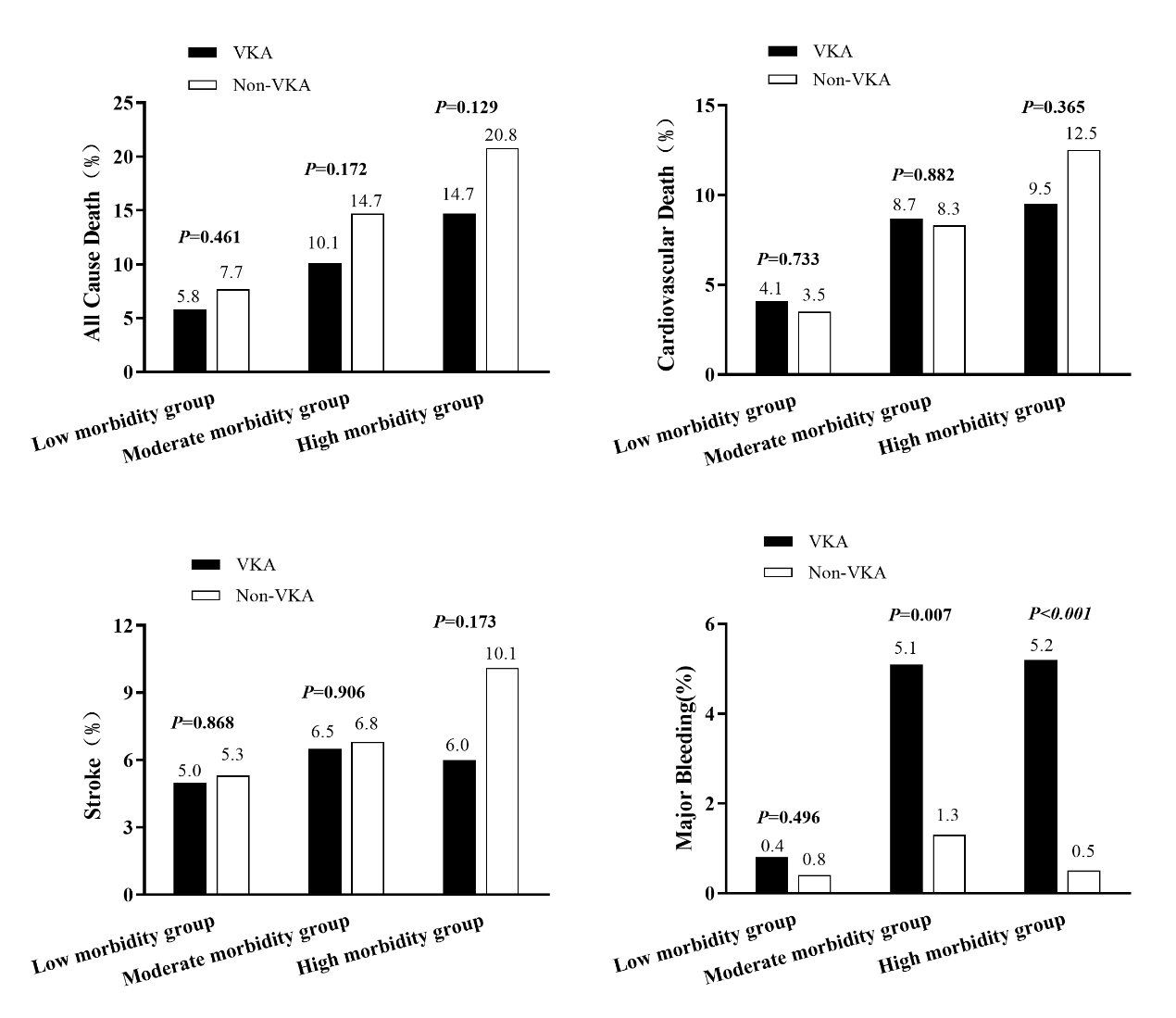


**Supplement Figure S6** Event rates of outcome variables in patients with AF by VKA or Non-VKA usage and morbidity groups


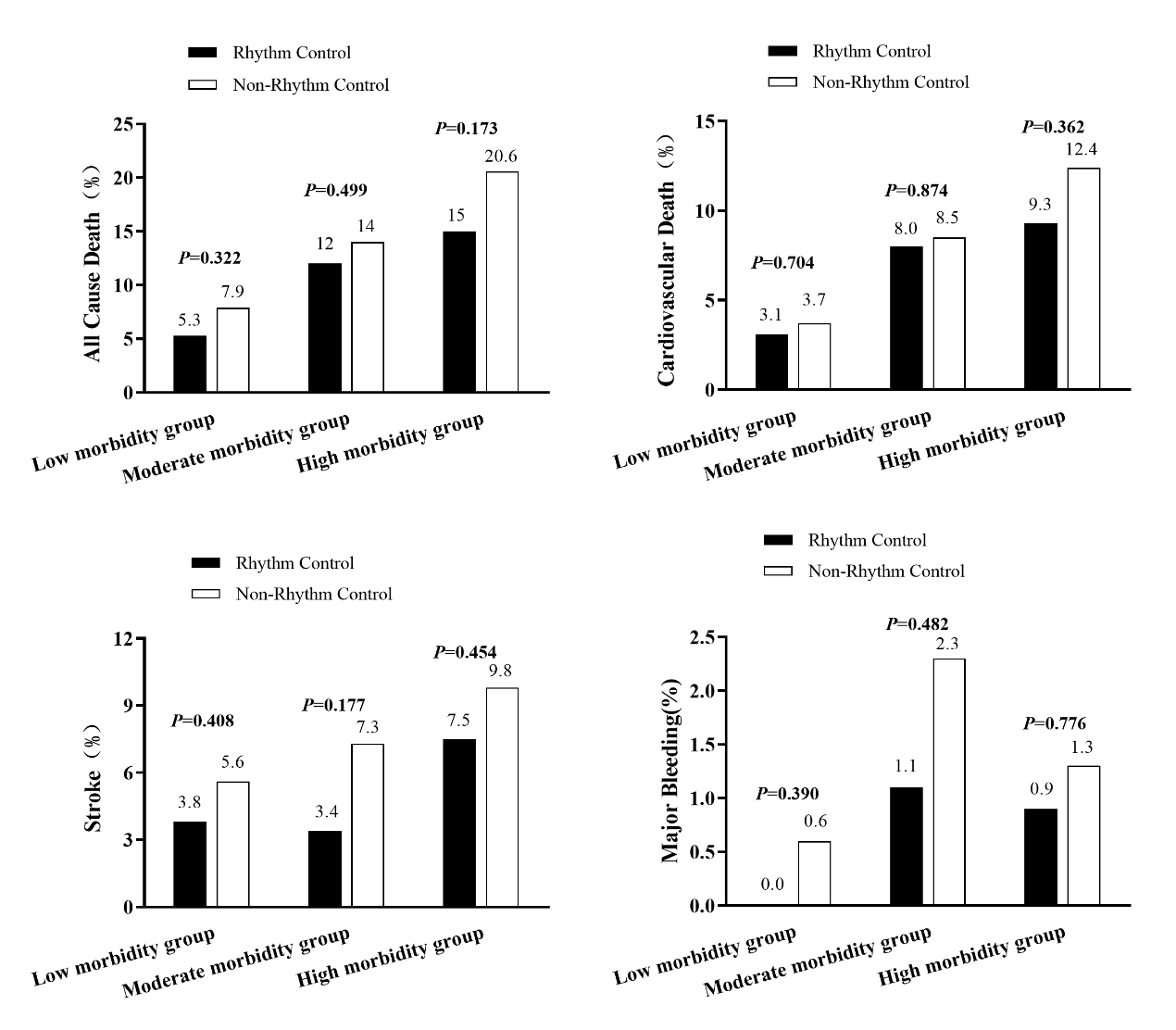


**Supplement Figure S7** Event rates of outcome variables in patients with AF by Rhythm Control or Non-Rhythm control strategy and morbidity groups


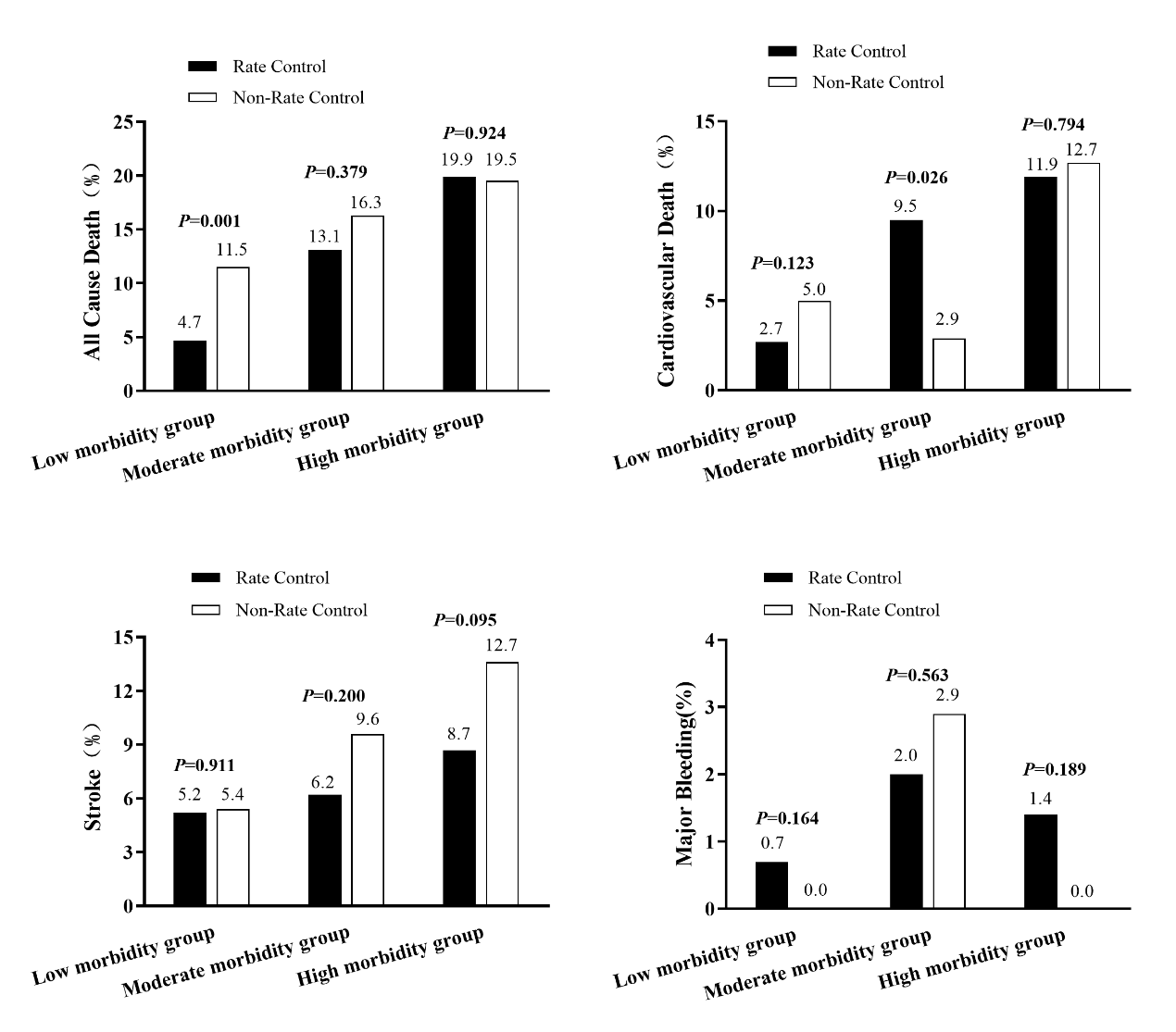


**Supplement Figure S8** Event rates of outcome variables in patients with AF by Rate Control or Non-Rate control strategy and morbidity groups


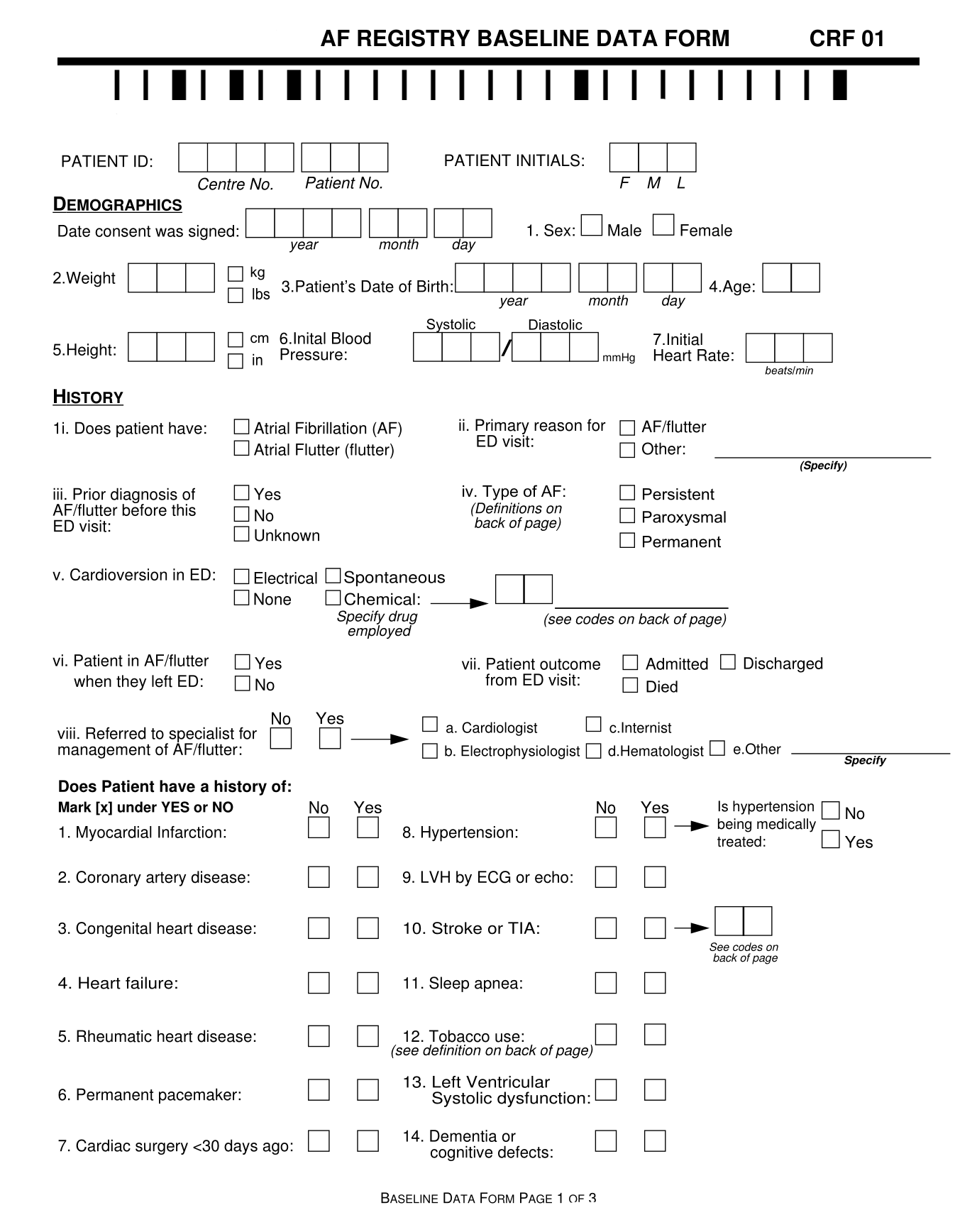


**Supplement Figure S9** Chinese emergency department AF registry CRF1


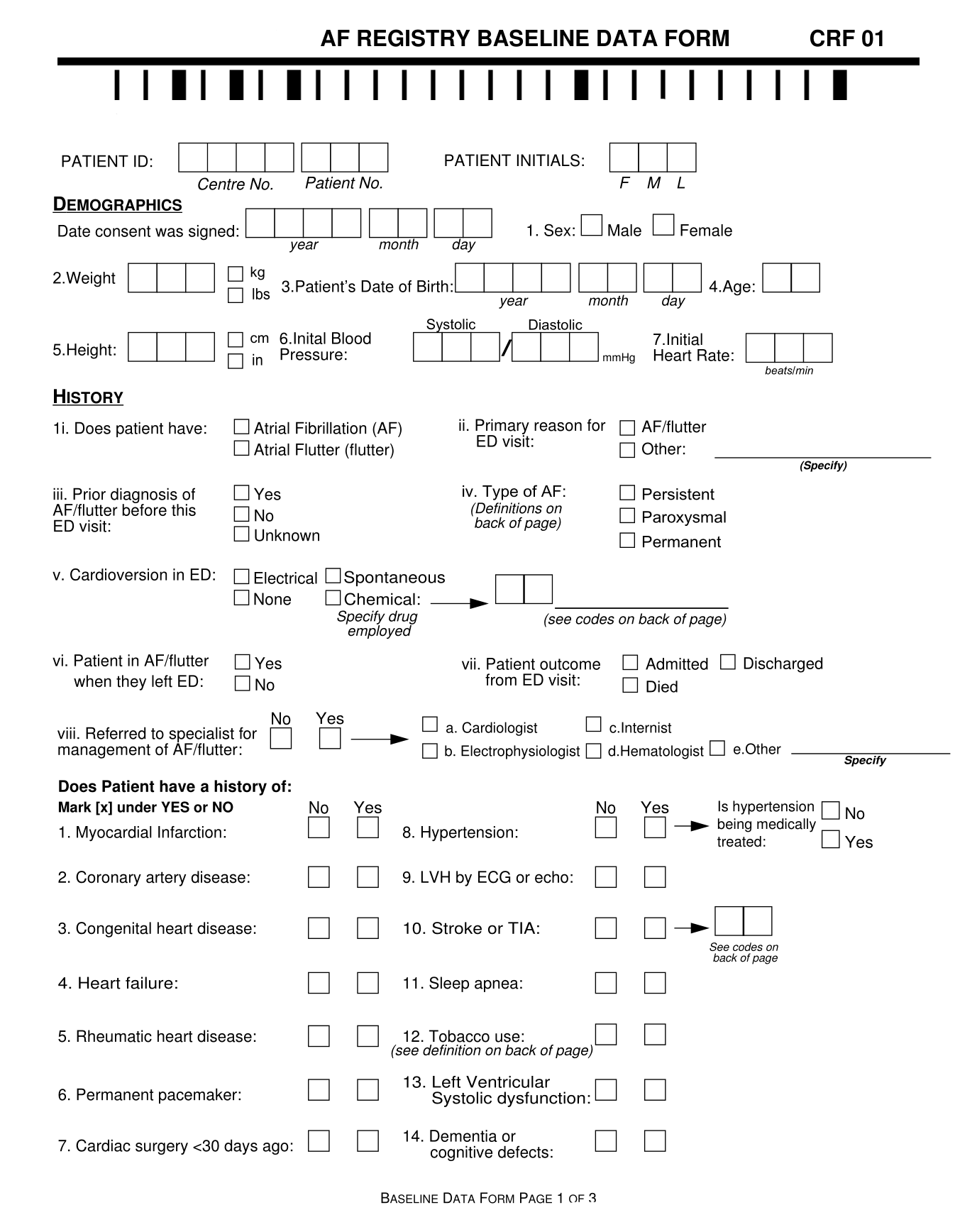


**Supplement Figure S10** Chinese emergency department AF registry CRF2
